# The effect of a trunk release maneuver on Peak Pressure Index, trunk displacement and perceived discomfort in older adults seated in a high Fowler’s position: a randomized controlled trial

**DOI:** 10.1186/1471-2318-12-72

**Published:** 2012-11-16

**Authors:** Krista L Best, Guylaine Desharnais, Jeanette Boily, William C Miller, Pat G Camp

**Affiliations:** 1 Rehabilitation Sciences Graduate Program, University of British Columbia, T325 - 2211 Wesbrook Mall, Vancouver, BC, V6T 2B5, Canada; 2 Banfield Pavilion, Vancouver Coastal Health, Ash Street, Vancouver, BC, Canada; 3 G F Strong Rehabilitation Centre, 4255 Laurel Street, Vancouver, BC, Canada; 4 Department of Occupational Science and Occupational Therapy, University of British Columbia, Vancouver, BC, Canada; 5 Department of Physical Therapy, University of British Columbia, Vancouver, BC, Canada; 6 James Hogg Research Centre, St. Paul’s Hospital, Vancouver, BC, Canada

## Abstract

**Background:**

Pressure ulcers pose significant negative individual consequences and financial burden on the healthcare system. Prolonged sitting in High Fowler’s position (HF) is common clinical practice for older adults who spend extended periods of time in bed. While HF aids in digestion and respiration, being placed in a HF may increase perceived discomfort and risk of pressure ulcers due to increased pressure magnitude at the sacral and gluteal regions. It is likely that shearing forces could also contribute to risk of pressure ulcers in HF. The purpose of this study was to evaluate the effect of a low-tech and time-efficient Trunk Release Manuever (TRM) on sacral and gluteal pressure, trunk displacement and perceived discomfort in ambulatory older adults.

**Method:**

A randomized controlled trial was used. We recruited community-living adults who were 60 years of age and older using posters, newspaper advertisements and word-of-mouth. Participants were randomly allocated to either the intervention or control group. The intervention group (n = 59) received the TRM, while the control group (n = 58) maintained the standard HF position.

**Results:**

The TRM group had significantly lower mean (SD) PPI values post-intervention compared to the control group, 59.6 (30.7) mmHg and 79.9 (36.5) mmHg respectively (p = 0.002). There was also a significant difference in trunk displacement between the TRM and control groups, +3.2 mm and −5.8 mm respectively (p = 0.005). There were no significant differences in perceived discomfort between the groups.

**Conclusion:**

The TRM was effective for reducing pressure in the sacral and gluteal regions and for releasing the trunk at the point of contact between the skin and the support surface, but did not have an effect on perceived discomfort. The TRM is a simple method of repositioning which may have important clinical application for the prevention of pressure ulcers that may occur as a result of HF.

## Background

Up to 65% of older Canadians and Americans develop pressure ulcers [1], which can lead to pain, fear and anxiety, isolation, reduced quality of life, and in some cases death [2,3]. The occurrence of pressure ulcers may increase the amount of professional healthcare needed, which may pose significant financial burden on the healthcare system [4]. According to Woodbury and Houghton, the prevalence of pressure ulcers in Canada is 25% in acute care, 30% in non-acute care, 22% in mixed health-care settings, and 15% in community care [5]. Currently there is no Canadian data that provides a cost analysis for treatment of pressure ulcers in residential facilities, but a Canadian cost estimate for the treatment of a stage three pressure ulcers in the community was approximately $9000 per patient/month [6].

A population at particular risk of pressure ulcers is older adults with complex care needs, who often also have limited mobility and activity tolerance that leads to greater periods of time spent in bed. In addition to resting and sleeping, the hospital bed becomes a place to engage in daily activities, such as eating, reading and socializing. Nursing textbooks and tradition recommend that patients in bed be positioned in a high Fowler’s position (HF) to optimize breathing, eating and conversation [7,8]. It is also recommended that patients moved into HF position for meals remain seated upright for 30 min afterwards to reduce the risk of reflux and aspiration [9].

**Figure 1:**
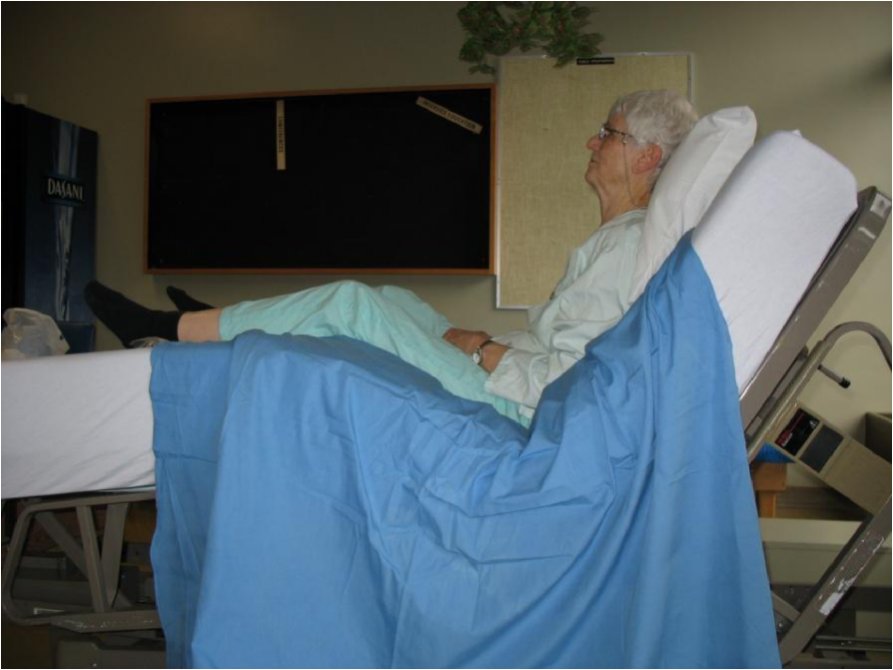
Older adult sitting in a high Fowler’s position.

HF is defined as a semi-upright position, in which the patient's head is raised 60 to 90 degrees (Figure 1) [10]. Despite the benefits of HF, the repercussions include increased feelings of perceived discomfort, and increased pressure at the sacral and gluteal regions [11]. Pressure over a bony prominence has been identified by the International Pressure Ulcer Guidelines [12] as a factor in the development of pressure ulcers, with the degree of risk related to magnitude and duration of pressure [7,13,14].

The guidelines also state that pressure combined with shear poses a significant risk for the development of pressure ulcers, which occurs when friction and opposing forces occur at a localised point. Reasoning would suggest that a patient is also at high risk of shearing forces when placed in HF, because the skin over the sacral region will be exposed to friction at the point where it comes in contact with the bed clothes, sheets, and mattress surface, when head of the bed is elevated and gravity exerts a downward pull on the body [15]. The shearing forces generated when the body is raised into HF are estimated to be ten times more likely to lead to ulcers compared to pressure alone [16]. Pressure ulcers occur if pressure and shearing are not alleviated, which is common in people who do not have the ability to independently reposition their body [17]. Interventions aimed at repositioning may offer a cost-effective and low-tech solution to reducing risk of pressure ulcers in frail older adults.

The impetus for this study arose when study investigators (GD, JB) noted that residents in a long-term care facility often expressed verbal and non-verbal manifestations of discomfort or refusal to be placed in HF. Clinical observations suggested that passive repositioning of the trunk immediately reduced discomfort and interface pressure at the sacral region, which lead to the development of a novel and low-tech Trunk Release Maneuver (TRM) to reposition the trunk while sitting in HF.

Our primary objective was to test the hypothesis that the TRM would significantly decrease pressure at the sacral and gluteal regions in older adults. Secondarily, we evaluated the effect of the TRM on trunk displacement (a proxy measure for shear) and perceived discomfort.

## Methods

### Study design

We conducted a randomized, controlled, single blind trial at a long term complex care facility in Vancouver, Canada. Recruitment occurred over a 13 month period (March 2010-April 2011). This RCT was registered with ClinicalTrials.gov (ID NCT00961012, Unique Protocol ID H09-10370) and was approved by the University of British Columbia Clinical Research Ethics Board and the Vancouver Coastal Health Research Institute. Participant consent to photography was obtained for all figures included in this manuscript.

### Participants

Due to the novelty of the TRM intervention and our clinical perception that HF places individuals with complex care needs at increased risk of pressure ulcers, we recruited a sample of ambulatory older adults who would be less at risk. A convenience sample of community-living older adults was recruited using posters and advertisements in senior-oriented residential buildings, community centers, seniors’ fitness centers, and local newspapers. Word-of-mouth and snowballing techniques were also utilized.

Participants were eligible for the study if they: were 60 years of age or older; able to speak English; able to give informed consent to participate; and had a Folstein Mini-Mental State Exam (MMSE) [18] score of 22 or higher [19], indicating that basic cognitive abilities were unimpaired. Participants were excluded from the study if they were at moderate to high risk for pressure ulcers, as determined by a score of 14 or less on the Braden Scale for Predicting Pressure Sore Risk.

Sociodemographic and personal information (age, sex, marital status, education, place of residence) and health-related variables (body mass index, MMSE, Functional Comorbidity Index, and the Braden Scale for Predicting Pressure Sore Risk) were collected at baseline prior to randomization. The Functional Comorbidity index is an 18-item list of diagnoses that uses a simple count (yes/no) to derive a score between 0 and 18, where 0 represents no comorbid illness and 18 represents the highest number of comorbid illnesses [20]. The Braden scale is a pressure ulcer risk assessment scale comprising 6 subscales. All subscales are ranked numerically from 1–4, except one that is ranked from 1–3. A summary score is derived by summing up the responses with scores ranging from 6–23. Lower scores indicate higher risk for pressure ulcer [21].

### Randomization

Participants were randomly allocated to either the intervention (TRM) or control group. The randomization sequence was developed using a computer generated table of random numbers by a biostatistician who was not associated with the study. Group allocation was concealed using individual sealed opaque envelopes that were numbered in sequential order. As individuals were enrolled in the study the next envelope in the sequence was extracted and the participant was assigned to the TRM or control group accordingly.

### Measurement

The primary outcome measure was interface pressure, measured as the Peak Pressure Index (PPI) in mmHg. Interface pressure was collected using an FSA torso pressure mapping system (Vista Medical, Winnipeg, Canada), which consisted of a sensing mat that was connected to an interface box, which relayed the information to a computer for real-time visualization and recording [22]. The FSA system is designed to characterize the magnitude and distribution of forces via the use of multiple sensels. The sensing mat was calibrated weekly using a standardized calibration device that consisted of 2 wooden platforms and an air bladder that can be inflated to a specified pressure. The sensing mat was calibrated to measure pressures between 0 to 200 mmHg. PPI was calculated by averaging the sensel with the highest pressure and the 3 surrounding sensels with the highest pressure according to the methods described by Sprigle, et al. [23]. PPI has been reported to be the most consistent measure of pressure magnitude [24], has excellent reliability [25], and is supported as an acceptable measure of pressure by the International Standards Organization (ISO) [26].

**Figure 2:**
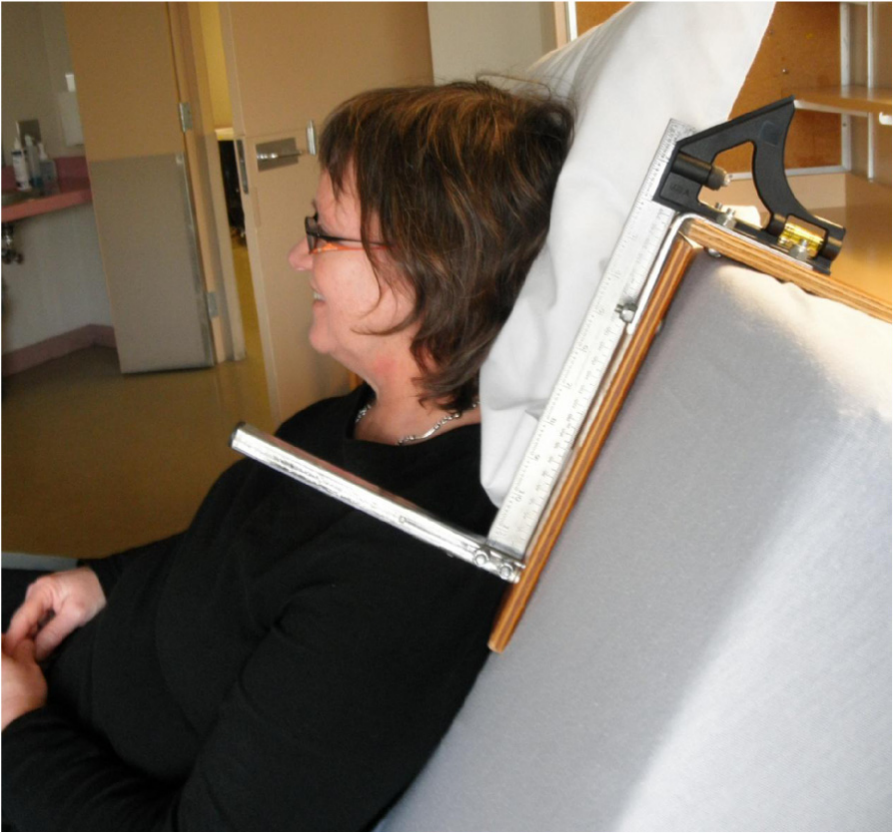
Trunk displacement was evaluated using a height gauge to measure the distance from the top of the mattress to the top of the shoulder.

Secondary outcomes measured included trunk displacement (a proxy measure for shear), which was used to measure whether the trunk had moved in relation to the surface of the mattress from the time of being placed in HF to after the intervention period. Changes in trunk displacement were used to determine whether the intervention could reset the relationship between the surface of the mattress and the trunk. Trunk displacement, defined as the change in the distance between the top edge of the mattress to the top of the participants’ shoulder (at the acromion process), was quantified using a height gauge [15]. The height gauge was comprised of a combination square that was fit with a spirit level to ensure consistent placement of the apparatus on the top of the mattress as shown in Figure 2. After being placed in HF, the tester placed the square-end of the height gauge across the flat part of the mattress and measured displacement to the top of the shoulder in millimetres (mm) to obtain a reference value for trunk position. A positive trunk displacement relative to the reference value indicated that the trunk had moved downward on the mattress surface. A negative trunk displacement suggested that the trunk had moved upward on the mattress surface relative to the reference value, suggesting that the frictional relationship between the trunk and mattress may have been reset. A resetting of the frictional relationship between the trunk and mattress is believed to be associated with a reduction in the amount of shear that may occur at the point where the skin contacts the mattress.

We also measured perceived discomfort using either: 1. a horizontal numeric scale (ranging from 0–10 in increments of 1) with word anchors (ranging from no discomfort to very much discomfort) or; 2. the Wong-Baker Faces scale, which consists of 6 faces displaying emotions that range from very sad to very happy and a numeric equivalent ranges from 0–10 in increments of 2 [27]. Participants were given the choice to use either the numeric scale or the Wong Baker scale, depending on their preference. Location of discomfort was obtained by asking the participants to point to a diagram of an outline of an anterior and posterior view of the human body. The specific areas where the participant felt discomfort were circled by the researcher on the diagram. Participants were told to choose all regions of the body in which they felt discomfort.

### Protocol

Two research assistants (RA1 and RA2) were trained by a study investigator. Each RA completed about 2 h of training on equipment set-up, screening procedures, data collection and administration of the intervention. The study investigator monitored procedures for the first 4 participants and did random checks thereafter to insure fidelity.

Data were collected in one 45–60 min session. RA1 screened subjects and obtained demographic information, self-reported Braden Pressure Ulcer Risk scores, and Functional Co-Morbidity Index from each participant. RA2, who was blind to group allocation, recorded personal information (height, weight, age, sex), and collected data for interface pressure (PPI), trunk displacement, and perceived magnitude and location of discomfort.

The study was conducted using a hospital bed that was fitted with a visco-elastic foam mattress, a fitted sheet, the FSA torso sensing mat positioned sideways under the participants’ buttocks, a thin protective plastic layer, a flat sheet and a pillow. All participants wore hospital pyjamas over their undergarments. Participants were then invited by RA1 to lay supine on the hospital bed with their pelvis centered on the sensing mat and with their hands resting on their abdomen. RA2 ensured proper placement of the pelvis on the sensing mat through real-time visualization provided on a computer. Adjustments to positioning were made as necessary. The participants were coached by RA1 throughout the study to remain completely immobile for the duration of data collection.

Once the participant was properly positioned in the bed, RA2 took one baseline measure of perceived magnitude and location of discomfort while the participant was lying supine. RA1 then placed the participant in HF by raising the foot of the bed first to its highest position (approximately 50 degrees) followed by the head of the bed to its highest position (approximately 60 degrees). The same hospital bed was used for all subjects. RA2 immediately measured trunk displacement and perceived magnitude and location of discomfort. After a wait period of 8 min, RA 2 recorded 3 consecutive pressure map images. The 8 min wait period was to allow for “creep” in the FSA pressure sensing mat and mattress. At this point RA 2 left the testing area for 3 min.

Group allocation was determined after enrolment into the study by RA1, who opened the sealed opaque envelopes in sequence. Participants who were allocated to the intervention group underwent the TRM, while participants in the control group were coached to remain still.

After the 3 min intervention or control period, RA2 returned to the testing area and immediately measured trunk displacement and perceived magnitude and location of discomfort in both the TRM and control group. All participants stayed in HF for an additional 8 min ‘creep’ period before RA2 took the final 3 consecutive pressure map images.

### Intervention - The Trunk Release Maneuver (TRM)

**Figure 3:**
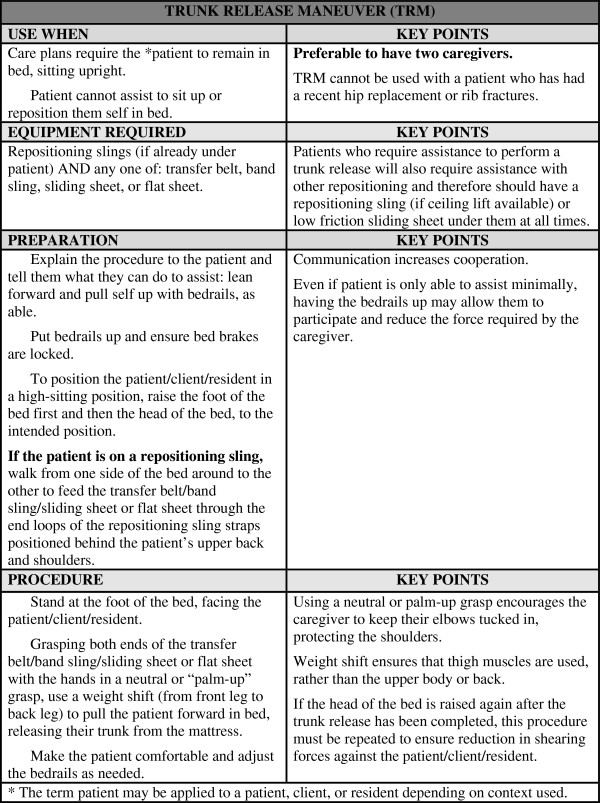
Description of the Trunk Release Maneuver.

The Trunk Release Maneuver (TRM) was developed by 2 of the study investigators with the assistance of the Musculo-Skeletal Injury Prevention (MSIP) team at Vancouver Coastal Health (Figure 3).

**Figure 4:**
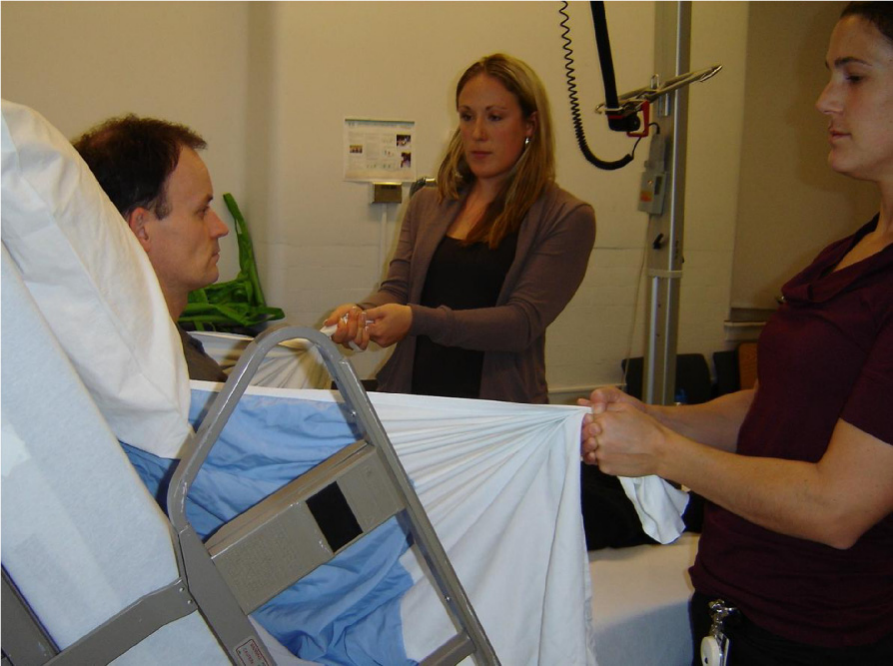
TRM being performed by 2 attendants.

The TRM is a standardized protocol that consists of pulling the trunk forward and away from the support surface of the bed without lifting the buttocks. The trunk can be pulled forward using either a positioning sling or a slider sheet. The trunk release can be performed by 1 or 2 attendants, as explained in Figure 3. For the purposes of this study the TRM was performed by 2 people (RA1 and GD) as shown in Figure 4.

*Control -* Participants in the control group did not receive any intervention. They remained positioned in HF position and were coached not to move by RA1.

Participants in both groups were coached not to speak about the intervention period when RA2 re-entered the data collection area.

### Statistical analysis

Descriptive statistics (mean, standard deviation, frequency) were calculated for all sociodemographic and personal characteristics. Differences for all variables at baseline between the intervention and control groups were analyzed using unpaired t-tests. Complete data were obtained for all participants who completed baseline measures; therefore, intention-to-treat analyses were not necessary. The primary objective to evaluate the effect of TRM on PPI was analyzed using Analysis of Covariance (ANCOVA), to control for differences between the groups at baseline in Functional Comorbidity Index. Within-subjects changes over time were analyzed using paired t-tests and relative change scores were calculated for both groups. Similarly, the secondary objectives (to evaluate the effect of TRM on change in trunk displacement and change in perceived discomfort) were analyzed using ANCOVA, to control for group differences at baseline in Functional Comorbidity Index. Within-subjects changes were analyzed using paired-t-tests. Bonferroni corrections were made to account for multiple comparisons using an adjusted alpha of 0.017 (0.05/3). As there was a significant difference between study groups in the numbers of comorbidities, all comparisons were adjusted for this covariate. All data were analyzed using IBM SPSS Statistics version 19.0.

### Power calculation

We required a minimum sample size of 120 subjects for this study. Due to the novel nature of this study there were no data on effect size or variance to permit a power analysis for any of the dependent variables of interest. To compensate for this we took 15 measurements of peak pressure index (PPI) from a single subject (Table 1). Given the lowest possible difference in the range of scores (64 mmHg before and 59 mmHg after trunk release) we anticipated a conservative effect size of 0.6 (expected difference/pooled standard deviation (SD)= 5 mmHg/8). Using an alpha of 0.01 and power of 0.80 we required 60 subjects per group for a two sided analyses [28]. A sample of this size also enabled us to detect a two unit difference [27] in discomfort (pooled SD of 2; alpha of 0.01; power 0.9). Trunk displacement is an exploratory construct with no similar known previous measurement data. Based on our pilot work a 4 cm displacement was achievable and with a pooled SD of 3 we required 34 subjects (17 per group) to reject the Null Hypothesis given an alpha of 0.01 and power of 0.8.

**Table 1:** Peak pressure index (PPI) before and after trunk release*

**Peak Pressure Index (PPI)**	**Before trunk release**	**After trunk release**
Range	64 to 98.75 mmHg	39.5 to 59 mmHg
Median	85.3 mmHg	52.3 mmHg

*These results are from a preliminary pressure mapping study that explored the influence of bed linen layers on interface pressure. High pressure over the sacral-coccygeal area was recorded when the person was moved into high Fowler’s position. The procedure was repeated 15 times with different bed linen layers.

## Results

### Study sample

**Figure 5:**
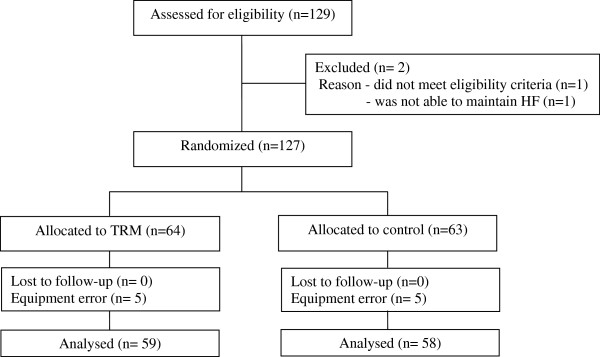
CONSORT diagram of progress through the enrolment, intervention allocation, follow-up, and data analysis of this study.

Figure 5 depicts the passage of participants throughout the study procedures (enrolment, intervention allocation, follow-up, and data analysis) as per the Consolidated Standards of Reporting Trials (CONSORT) statement ( http://www.consort-statement.org). A total of 129 participants were enrolled into the study. Two participants were excluded from the study because one did not meet the inclusion criteria and one experienced unbearable neck pain due to an existing injury when placed in HF. A total of 127 participants were randomized into the TRM (n = 64) or the control (n = 63) group. Due to equipment malfunction with the pressure map, data for 5 participants from each group were excluded from the analysis. Complete data were obtained and analyzed for a total of 117 participants, (TRM = 59 and control = 58).

### Sociodemographic and personal information

The participants in this study ranged in age from 60–88 years, with a mean (standard deviation (SD) age of 67.4 (6.7) years. The sample was comprised of predominantly single (65%), females (62%) participants, of whom 96% were educated at the post-secondary level (Table 1). The participants were physically healthy, with a mean (SD) Body Mass Index (BMI) of 24.8 (4.5) kg/m^2^; Functional Comorbidity Index of 2.2 (1.6), and were cognitively high functioning with a Mini Mental State Exam score of 29.3 (1.1). Group equivalence was achieved on all sociodemographic and personal variables except the intervention group had a significantly higher number of comorbidities [Functional Comorbidity Index = 2.5 (1.8)] compared to the control group [Functional Comorbidity Index = 1.8 (1.3)]. Sociodemographic and personal information are summarized in Table 2.

**Table 2:** Sociodemographic, personal and health-related variables

**Participant Characteristics**	**Sample**	**Control**	**TRM**	**p-value**
**Demographics**	**n = 117**	**n = 58**	**n = 59**	
Age, y, mean (SD)	67.4 (6.7)	67.4 (6.7)	68.5 (6.3)	0.63
Sex, no. (%)				0.11
Male	27 (23.1)	17 (29.4)	10 (16.9)	
Marital Status, no. (%)				0.57
Married/Common Law	45 (38.5)	21 (36.2)	24 (40.7)	
Widowed/Separated/Divorced	44 (37.6)	21 (36.2)	23 (38.0)	
Single	28 (23.9)	16 (27.6)	12 (20.3)	
Education, no. (%)				0.40
High School (or less)	21 (17.9)	13 (22.4)	8 (13.6)	
College/Trade School/University	96 (82.1)	45 (77.6)	51 (86.4)	
Health-Related Variable, mean (SD)				
BMI (Kg/m^2)	24.8 (4.5)	25.1 (4.3)	25.6 (4.6)	0.23
MMSE (/30)	29.3 (1.1)	29.1 (1.3)	29.4 (0.8)	0.01
Braden Pressure Ulcer Risk (/23)	22.8 (0.4)	22.9 (0.4)	22.8 (0.4)	0.03
Functional Comorbidity Index (/18)	2.2 (1.6)	1.8 (1.3)	2.5 (1.8)	*0.04

* Significant difference between groups.

### Peak Pressure Index (PPI)

There were no significant differences in mean (SD) PPI values between the TRM and control groups at baseline, 71.3 (37.8) mmHg and 76.8 (35.5) mmHg respectively. The TRM group had significantly lower mean (SD) PPI values post-intervention compared to the control group, 59.6 (30.7) mmHg and 79.9 (36.5) mmHg respectively [F(1,114) = 9.76, 95% CI = 7.32, 32.67, p = 0.002].

**Figure 6:**
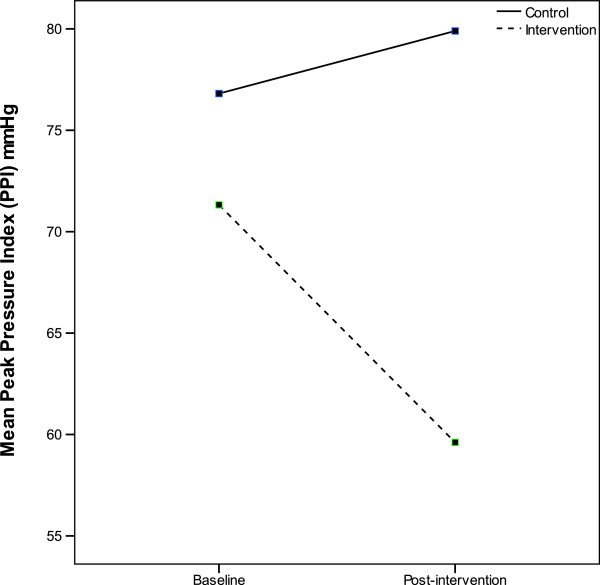
**Change in peak pressure index (PPI) from baseline to post-intervention in the TRM and control group.** The TRM had a significant decrease in of 11.7 mmHg compared to the control group who significantly increased by 3.1 mmHg.

Within-subject analysis showed the mean (SD) PPI was significantly reduced by 11.7 (16.2) mmHg in the TRM group from baseline to post-intervention [p < 0.001, 95% CI = −15.9, -7.5]; whereas the control group had a mean increase in PPI of 3.1 (7.1) mmHg over the same time period [p = 0.002, 95% CI = −4.9, -1.2]. Figure 6 shows between-group and within-subjects comparisons of PPI between the TRM and control group.

### Trunk displacement

**Figure 7:**
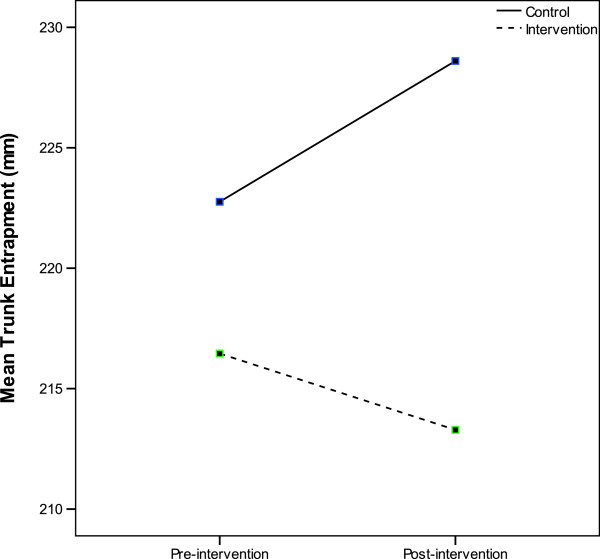
**Change in trunk entrapment from baseline to post-intervention in the TRM and control group.** The amount of trunk entrapment differed significantly by 9 mm between the TRM and control groups.

There was a significant difference in trunk displacement between the TRM and control groups post-intervention [p = 0.005, 95% CI = 4.2, 13.7] as seen in Figure 7. After receiving the intervention, the TRM group had a mean (SD) negative trunk displacement of 3.2 (15.5) mm relative to the supine position. Participants in the control group had a mean (SD) positive trunk displacement of 5.8 (9.0) mm, relative to the supine trunk position.

### Perceived discomfort

Both the TRM and control groups reported a significant increase in perceived discomfort when moved from lying supine to a high Fowler’s position. However, there were no significant differences in discomfort between the TRM and control groups while lying supine, immediately after being placed in a high Fowler’s position, or immediately post intervention.

### Adverse effects

One participant had existing neck pain that was exacerbated by HF. There were no adverse effects of the TRM for the participants or the researchers who administered it.

## Discussion

The results from this study supported our primary hypothesis that a novel Trunk Release Maneuver (TRM) to reposition the torso in HF lead to a reduction in interface pressure at the sacral and gluteal regions. The TRM group showed statistically significant reductions in PPI from baseline to post-intervention compared to the control group who had a statistically significant increase in PPI.

Interface pressure mapping is commonly used as a clinical tool to monitor potentially concerning areas of high pressure when sitting on various support surfaces, such as mattresses and wheelchair cushions. In combination with additional considerations, including skin condition, anatomical location, age, hydration, and metabolism of the individual, pressure mapping can help identify areas at risk for pressure ulcers.

While there is an association between interface pressure and risk of pressure ulcers [13], there is no solid evidence of a clinically significant pressure threshold. Current clinical practice guidelines suggest that efforts be made to reduce the duration and magnitude of pressure on areas of the skin that are in contact with a support surface [29]. Although we are not certain of a clinically significant pressure threshold, it is promising that participants in the intervention group had statistically significant reduction of 15 mmHg in mean PPI the sacral and gluteal regions after receiving the TRM compared to the control group who had a statistically significant increase in mean PPI. Since bony locations on the body are more prone to pressure ulcers [30], even slight reductions in pressure at the sacrum and ischial tuberosities may have clinical implications. Furthermore, relieving pressure magnitude at areas where shear force is present is even more important to reducing risk of pressure ulcers because pressure magnitudes that increase risk of pressure ulcers are almost half of that when little to no shear is present [31,32]. More research is needed to establish pressure thresholds and to determine clinically significant pressure reductions.

Previous studies provide support for interventions that reposition the body while lying in bed to reduce pressure magnitude [13,33,34]. A recent Cochrane Review stated that repositioning of the body is internationally recognized and promoted as an integral component of effective pressure ulcer management [35], especially for individuals who are unable to move themselves. Despite clinical recommendations and research findings that support the use of repositioning for the prevention of pressure ulcers, the optimal technique has not been determined [13,36]. The limited documentations on optimal repositioning techniques combined with clinical concern for the development of pressure ulcers in older adults who are not capable of independent repositioning lead to the development of the TRM. However, the exact mechanism behind the TRM and pressure reduction at the sacral and gluteal regions is not clear. It is thought to be a combination of the simple repositioning in the bed to redistribute pressure from the sacral and gluteal area to the entire torso, in addition to a release of the trunk from the frictional forces that occur where the skin over the sacral area contacts the support surface.

The notion of a release of the trunk from the friction that occurs where the sacrum and low back contact the support surface was supported by our findings that participants in the TRM group had negative trunk displacement after the intervention compared to participants in the control group who showed positive trunk displacement. It was thought that if the TRM was successful in resetting the frictional relationship at the point where the skin and mattress were in contact at the sacral region, the trunk would be released from the frictional forces and the individual would be sitting higher in the bed. Although we did not have a direct measure of shear, there is reason to believe that a positive trunk displacement would indicate more risk of shear at the point where the skin contacts mattress as a result of friction and opposing forces between the elevation of the bed and gravity acting on the body. If this is true and shear occurs at the specific point of contact, the resultant forces are 10 times more destructive to the skin than pressure alone [16]. Recognizing the limitations to our crude proxy measure for shear, shearing forces pose a major risk factor for pressure ulcers and have not been quantified in a clinical setting in the literature to date.

Surprisingly, there were no differences between the TRM and control groups for level of perceived discomfort. While some participants in the intervention group reported an improvement in discomfort after the TRM, other participants did not. This finding was similar for the control group. Participants in this study were generally healthy with a good BMI and healthy muscle mass. It is possible that a healthy muscle mass may contribute to the decreases sensation of discomfort overtime. Another possible reason for the lack of difference in perceived discomfort between the TRM and control groups was the use of a generic pain scale to quantify discomfort. Interestingly, although asked specifically about discomfort, many participants used the term pain when asked to rate their discomfort. Discomfort and pain are different constructs and pain measures may not have captured the more diffuse symptoms of discomfort that our participants experienced. It is possible that perceived comfort may have been a more suitable and responsive measure for this study. More research in this area is needed to differentiate these constructs.

The limitations of our study include the generalizability of our findings from a healthy population who did not have the complex care needs and health disparities that are often present in older adults in long-term care. However, we still observed a difference in PPI between the TRM and control groups. Given that our participants likely had a healthier muscle mass than what would be observed in residents of long-term care facilities, it is plausible that even greater differences in PPI would be observed in a more vulnerable population. The challenge in determining the location of PPI should also be noted. Due to measurement of PPI on an FSA torso mat, the location of PPI could be located anywhere over the area of contact. Moreover, the location of PPI may change between laying supine and being positioned in HF, or after performing TRM. Future studies on the TRM should consider palpation or other techniques to determine location of PPI.

The measurement limitations in our study included a crude indicator of trunk entrapment to capture displacement of the trunk. Although our measurement device was fitted with a square-gauge and level to level to increases consistency, the reliability and validity of this measure was not determined. The premise for measuring trunk entrapment came about as a proxy measure for shear. However, a precise measure of shear is needed to make any conclusion about the effect of TRM on the reduction of shearing forces. Other limitations arose from our inability to ensure our participants remained completely still. Although participants were coached not to move, they were often observed making small movements of the limbs which may have influenced pressure and trunk entrapment measurements. Finally, we did not continue to measure pressure readings over several hours. It is unknown if prolonged sitting time in HF will negate the benefits of performing the TRM. However, it is known that prolonged sitting in an upright position is not advisable from a pressure ulcer prevention point of view. Therefore, it is suspected that frequent repositioning according to the TRM protocol would be necessary to reduce the risk of pressure ulcers.

## Conclusions

Placing healthy older adults in HF causes concerning pressure magnitudes over the sacral and gluteal regions. A novel, simple, and time efficient Trunk Release Maneuver to reposition the body reduces interface pressure and trunk displacement in the short term, but does not reduce perceived discomfort.

## Competing interests

None of the authors have competing interests to report.

## Authors’ contributions

All authors read and approved the final version of the manuscript. GD and JB conceived the project and developed the intervention. GD, JB, WM and PC developed the study hypotheses, protocol and wrote the grant to obtain funds for the project and assisted with interpretation of the findings. KB conducted the statistical analysis under the supervision of WM and PC. KB drafted the manuscript under the supervision of PC. KB provided assistance with collecting the study data.

## Pre-publication history

The pre-publication history for this paper can be accessed here:

http://www.biomedcentral.com/1471-2318/12/72/prepub
